# E-cigarette or Vaping Product Use-Associated Lung Injury Presenting as Sub-Acute Hypoxemia Without Increased Work of Breathing

**DOI:** 10.7759/cureus.9855

**Published:** 2020-08-18

**Authors:** Harmanpreet Chawla, Thomas Weiler

**Affiliations:** 1 Pediatrics/Critical Care, Presbyterian Hospital, Albuquerque, USA

**Keywords:** e-cigarettes, evali, vaping, lung injury, respiratory failure

## Abstract

A 15-year-old male with a history of mild intermittent asthma presented with fatigue, coughing, vomiting and anorexia which was progressive in nature over the past month. The patient was found to have evolving chest x-ray infiltrates bilaterally, and hypoxemia without accessory muscle use. The patient was placed on non-invasive continuous positive airway pressure (CPAP) for hypoxemia. A CT scan showed bilateral infiltrates in a pattern consistent with E-cigarette or Vaping product use-Associated Lung Injury (EVALI). Upon further history taking, the patient admitted to electronic cigarette (e-cigarette) use with a new tetrahydrocannabinol (THC) cartridge prior to the onset of symptoms. The patient was started on methylprednisolone after which his oxygen requirement improved. This case highlights the need to continue to be diligent regarding the use of e-cigarettes in the pediatric population, and be aware of the constitutional symptoms that are associated with their use.

## Introduction

The rising popularity of electronic systems of inhalation, electronic cigarettes (e-cigarettes) and vaping, was first introduced to United States market in 2006, and has resulted in the recognition and rising incidence of E-cigarette or Vaping product use-Associated Lung Injury (EVALI). These products have been marketed towards adolescents specifically. Initiatives for marketing towards the adolescent groups have included (but are not limited to) offering appealing flavors within their products, social media targeting campaigns as well as sponsoring various events. As a result, the pediatric population has been disproportionately affected by this emerging syndrome [1]. According to surveillance reports of pediatric patients (age 13-17 years) to the Centers for Disease Control and Prevention (CDC), 56% of patients with EVALI required admission to the intensive care unit, with 29% of those patients requiring intubation and mechanical ventilation [2]. Although the age group of adolescents and young adults (age 13-24) is approximately 20% of the general population in the United States, this same age group accounts for approximately 50% of all EVALI cases reported through the CDC [3].

With the increased use of e-cigarette devices, there now is a need for a high index of suspicion for their use, and an understanding of what the clinical syndrome entails. EVALI has a non-specific presentation, with non-pulmonary signs including fever, headache, dizziness, nausea, vomiting and diarrhea. Standard physical examination findings such as tachycardia and tachypnea are only seen in approximately half of patients. Vital signs should routinely include pulse-oximetry as 57% have been found to have an oxygen saturation <95% on room air. Admission therefore needs to be considered for those who have decreased oxygen saturation on room air, respiratory distress, or for those who have a baseline compromise to lung function [4].

## Case presentation

**Figure 1:**
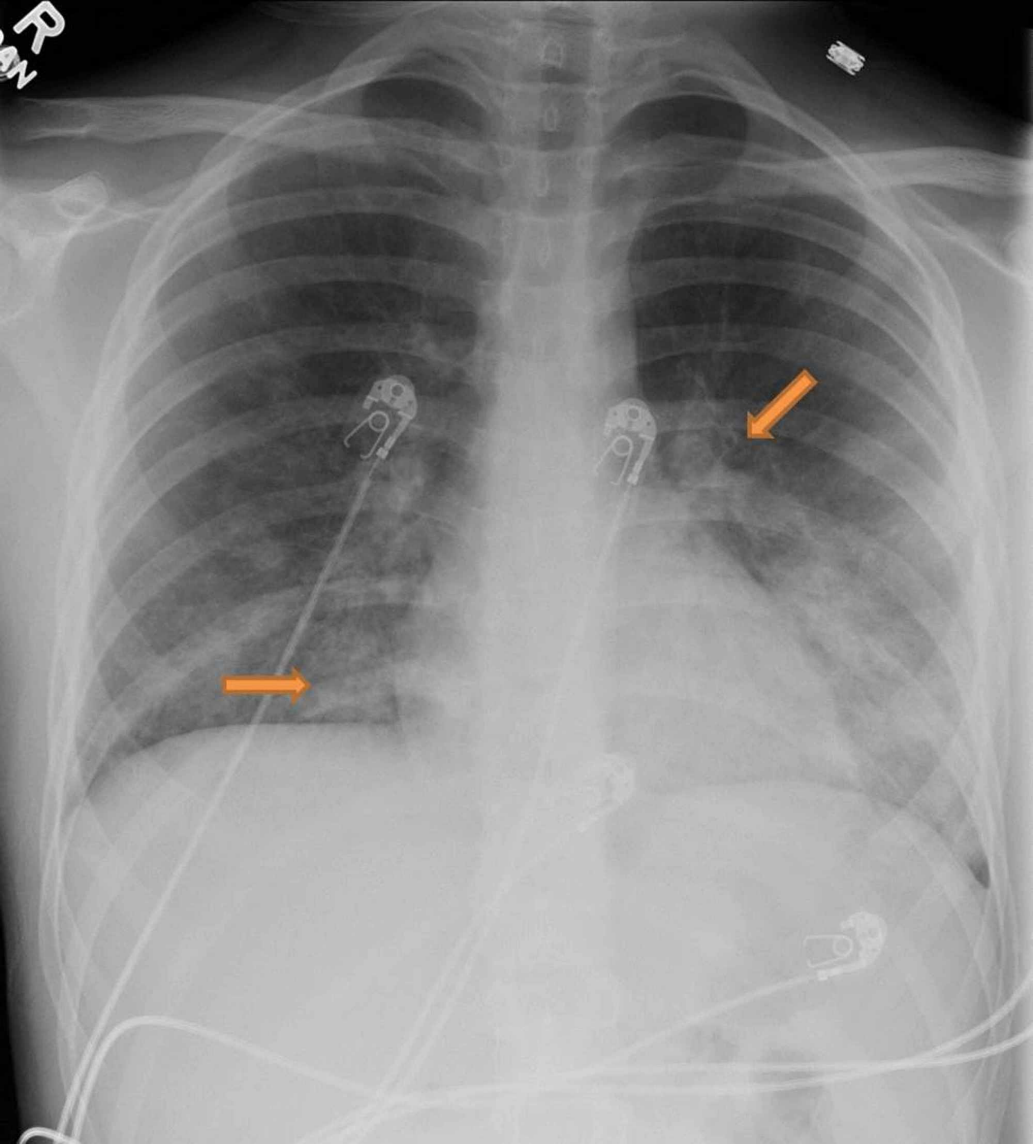
Left perihilar and right lower lobe basilar infiltrates most consistent with multifocal bronchopneumonia/atypical pneumonia.

The patient is a 15-year-old male with a past medical history of well controlled, mild-intermittent asthma who presented with a subacute onset of fatigue, cough, and vomiting. The patient was in his usual state of health until one month prior to admission when he developed fatigue and non-bloody, non-bilious emesis. Symptoms progressed with the development of anorexia, intermittent low-grade fevers (to a maximum of 102 F), cough, and shortness of breath, which was unresponsive to albuterol. Two days prior to admission, the patient was seen by his primary care provider who diagnosed him with atypical pneumonia based on history, physical exam, and chest radiograph (Figure 1). He was started on azithromycin.

**Figure 2:**
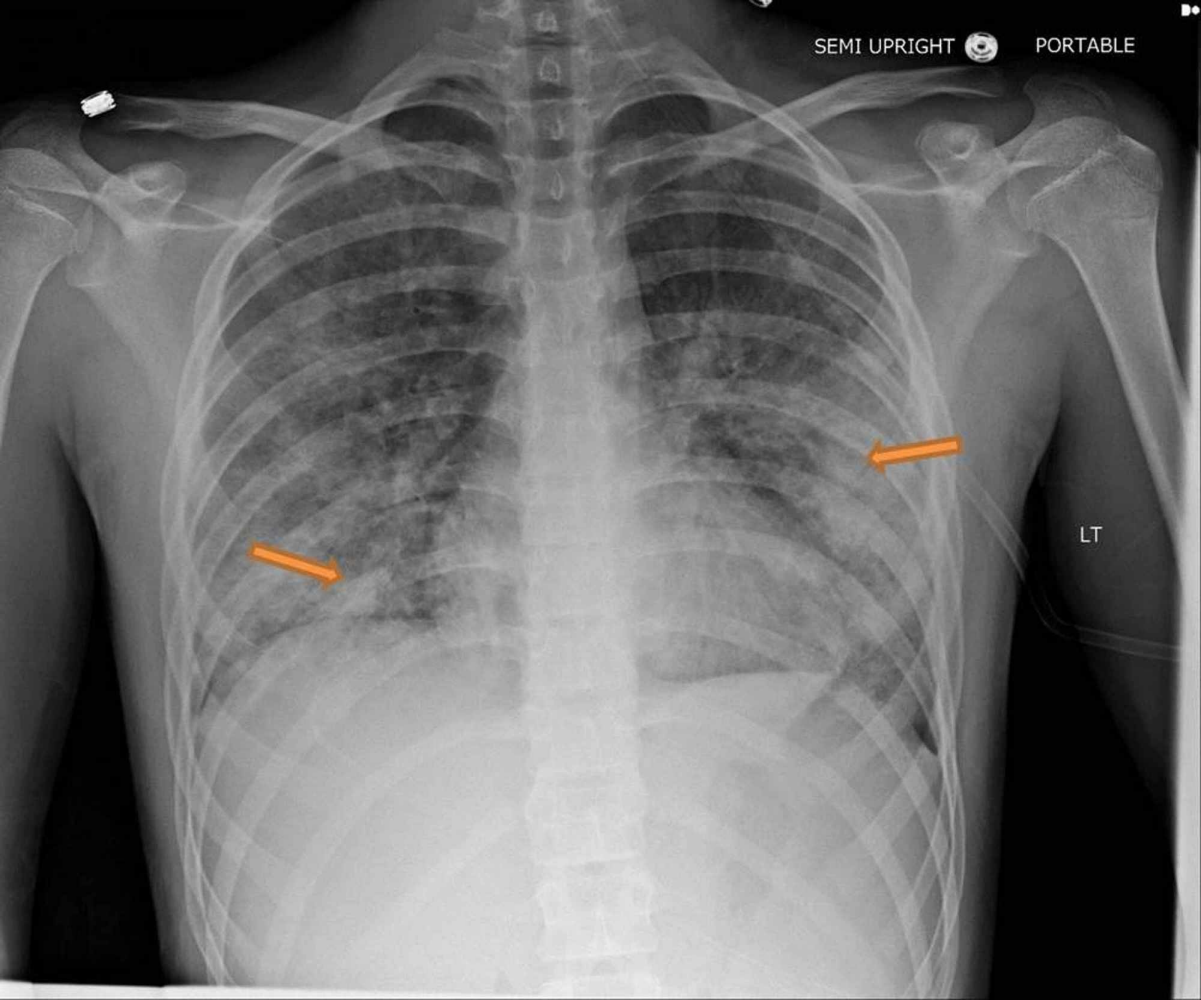
Worsening bilateral pulmonary opacity, with worsening patchy areas of consolidation involving the right lower lobe, the left mid lung field and now the upper lobes as well, right worse than left.

After two days of treatment with azithromycin the patient developed worsening shortness of breath and was brought into the emergency department where he was found to have tachycardia (with heart rates between 100-130), diminished aeration to auscultation, and hypoxemia in the absence of accessory muscle use. He was started on a non-rebreather facemask for his hypoxemia, however his saturations remained marginal at 90%. Chest radiograph showed disease progression (Figure 2) from the imaging two days prior. 

Due to his high oxygen requirement, the patient was admitted to the pediatric intensive care unit (PICU) where he was escalated to non-invasive continuous positive airway pressure (CPAP), requiring an FiO2 of 1.0. He continued to show mild tachypnea (respiratory rate 20-30 breaths/minute) and dyspnea (able to speak in four- to five-word sentences), with no accessory muscle use. The differential diagnosis for subacute shortness of breath in an adolescent patient was broad. Laboratory studies were notable for leukocytosis, elevated inflammatory markers (C-Reactive Protein/Procalcitonin) and elevated liver enzymes. Laboratory results are summarized in Table 1. The preliminary workup in this case failed to yield a diagnosis.

**Table 1:** Laboratory testing, including testing to rule out alternative etiologies of hypoxemic respiratory failure. AST: aspartate aminotransferase; ALT: alanine aminotransferase; BAL: bronchoalveolar lavage; PCR: polymerase chain reaction; Ig: immunoglobulin

Test	Result	Reference Range
White Blood Cell Count ( X10^3^/uL)	13.8	4-11
Hemoglobin (g/dL)	12.5	13.5-17.7
Hematocrit (%)	38	37-49
Platelets ( X10^3^/uL)	351	150-400
Neutrophils (%)	83	40-60
Lymphocytes (%)	9	20-40
Eosinophils (%)	5	0-7
Albumin (g/dL)	2.1	3.4-4.7
AST (U/L)	179	6-38
ALT (U/L)	187	13-63
C-Reactive Protein (mg/dL)	23.4	<0.3
Procalcitonin (ng/dL)	0.46	<0.10
(1,3) beta D glucan (pg/mL)	<31	<59
HIV Screen	Negative	-
Respiratory Viral Screen	Negative	-
Acid-fast bacilli (AFB) Culture	Negative	-
Pneumocystis jiroveci Smear	Negative	-
Mycoplasma PCR	Negative	-
Fungus Culture (BAL)	Negative	-
Chlamydia Panel (IgM/IgG)	Negative	-
Quantiferon Gold	Indeterminate	NR
Purified Protein Derivative (PPD) Skin Test	Negative	-

**Figure 3:**
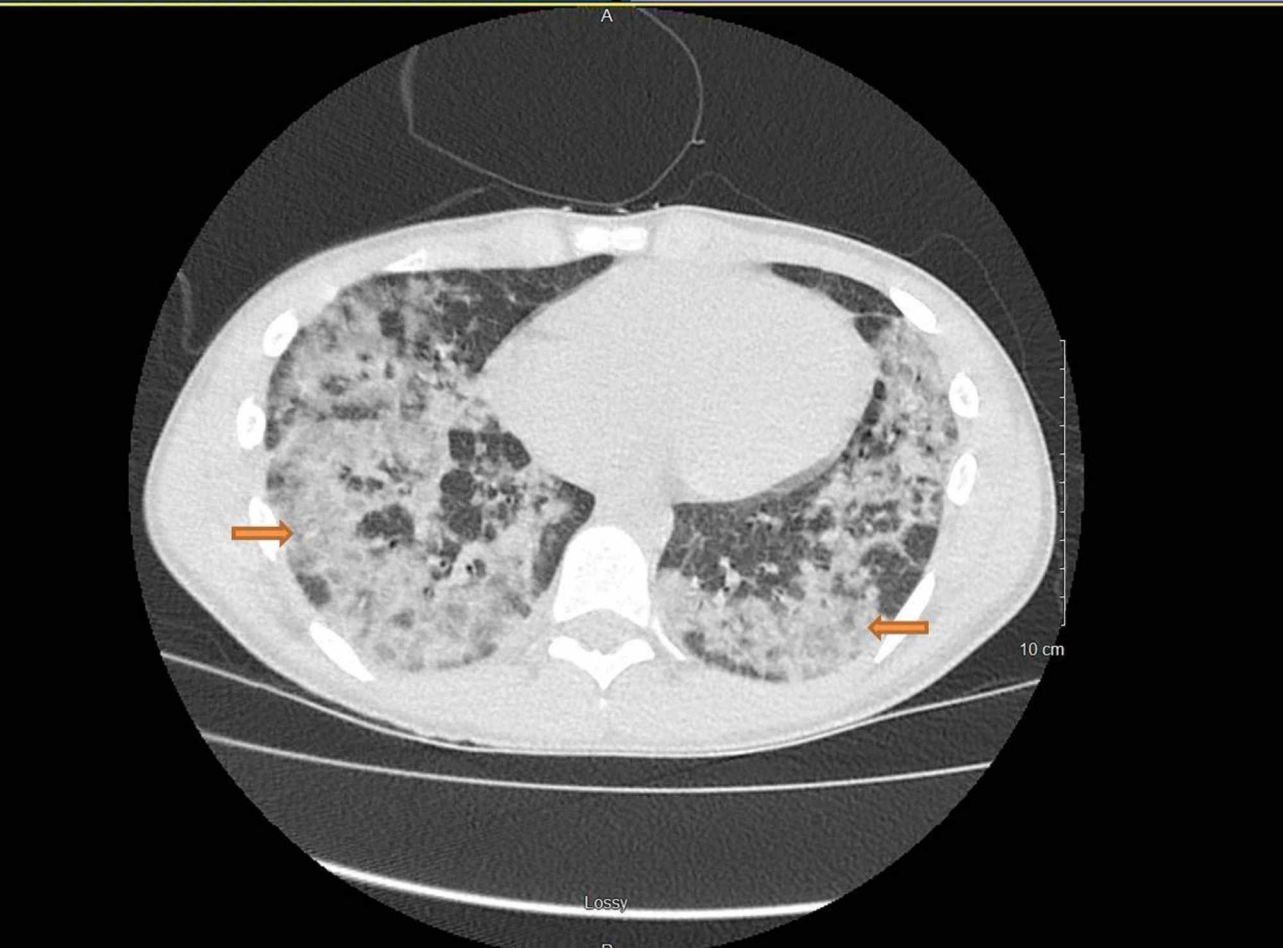
Bilateral and symmetric, basilar predominant airspace disease.

Despite his underlying diagnosis of asthma, the patient’s presentation was inconsistent with an asthma exacerbation (due to the absence of wheezing, prolonged expiratory phase, increased work of breathing, or response to beta-agonist therapy). A nasopharyngeal swab for respiratory viral polymerase chain reaction (PCR) was negative and he had no radiographic or laboratory findings consistent with pneumonia from community-acquired organisms, atypical organisms, or *Pneumocystis carinii* (of special interest due to his hypoxemia in the absence of increased work of breathing). Screening labs with immunoglobulins and T-cell subsets also failed to demonstrate any immunodeficiency. For further diagnostic evaluation, he underwent a non-contrast computerized tomography (CT) scan of the chest (Figure 3). The chest CT demonstrated non-specific bilateral, symmetric airspace disease which has been traditionally associated with acute alveolar hemorrhage, non-cardiac pulmonary edema, or viral pneumonia (none of which were supported by the other diagnostic evaluations). However, this pattern of pneumonitis has more recently been recognized to be associated with the sequelae of e-cigarette use in adult patients. After the findings of the CT chest raised this possibility, further history taking found that the patient did start using e-cigarettes approximately two weeks prior to the onset of his illness. Furthermore, he used a tetrahydrocannabinol (THC) cartridge (which has been implicated in more severe presentations of this syndrome [4]) immediately prior to the onset of his illness. These findings confirmed the diagnosis of EVALI.

The patient in this case received respiratory support with alternating Bilevel Positive Airway Pressure (BiPAP), CPAP and high flow nasal cannula for six days. He received intermittent nebulized hypertonic saline, to aid with his large secretion burden, and albuterol, given his history of asthma. As suggested by the existing literature, he was started on methylprednisolone (1mg/kg/dose q6 hours) for five days and subsequently, prednisone for two additional days. Additionally, he was started on empiric ceftriaxone and azithromycin (for five and seven days, respectively) for presumed community-acquired pneumonia. After his hypoxemia improved, a bronchoscopy and bronchoalveolar lavage was performed on hospital day six with negative cultures of bronchial alveolar lavage and unremarkable cellular cytology, showing only neutrophils and lymphocytes without hemosiderin-laden macrophages. 

## Discussion

Typically, affected patients have an indolent progression of their illness over weeks, culminating in dyspnea, cough, chest pain and hypoxemia. The underlying mechanism of EVALI is consistent with chemical pneumonitis and diffuse alveolar injury [3], secondary to the toxic substances within e-cigarettes including flavorants, vitamin E, and heavy metals; in addition to the more insidious and well-defined toxic effects secondary to nicotine- and cannabis-based products. THC-containing e-cigarettes have the ability to produce diffuse alveolar damage, as well as marked hyperplasia of type 2 pneumocytes [5]. 

A well-defined clinical syndrome is yet to be established. The Centers for Disease Control and Prevention (CDC) notes that tachycardia and tachypnea are only present in approximately half of the patients presenting with EVALI. The CDC recommends a chest radiograph on all patients with a history of e-cigarette use who have respiratory or gastrointestinal findings, especially when accompanied with hypoxemia. Laboratory testing on the adult populations typically reveals leukocytosis, elevated inflammatory markers, and elevated liver enzymes [4]. In a case study that examined both chest radiography and CT images of pediatric EVALI patients, 100% of patients were found to have bilateral symmetric ground-glass opacities. Consolidations were found on 57% of chest radiographs, and 64% of chest CT scans, with predominance for lower lobes on CT [6].

Optimal management of patients for EVALI is not yet well described. To date, there have been no randomized control trials evaluating therapeutic options. The few case series available to date have described medical management with corticosteroids as these are felt to attenuate the inflammatory response associated with EVALI. Case reports have noted clinical improvement coinciding with the initiation of corticosteroid therapy, however, there are no randomized controlled trials to support this practice. In a case series of adolescent patients, all patients showed an improvement in oxygenation parameters (S/F ratio) with initiation of methylprednisolone treatment [7]. Additionally, the concurrent use of antimicrobial therapy for community-acquired pneumonia and anti-viral therapy (in the setting of influenza) should be employed as indicated [8].

As more data is collected on EVALI, the lung injury does appear to have a reversible component. On follow-up for patients who have been diagnosed with EVALI, those who had cessation of vaping and had corticosteroids administered showed resolution of chest CT findings as well as normal spirometry measurements [9].

## Conclusions

Hypoxemic respiratory failure is a common reason for admission to the pediatric intensive care unit and with the growing prevalence of e-cigarette use, EVALI needs to be considered in the differential diagnosis of this problem. Additionally, patients with chronic constitutional symptoms also need to be asked regarding EVALI use. A thorough history from the patients or their families should include not only an inquiry about the use of e-cigarettes or vaping, but also about the content of the cartridges. The presentation for EVALI can be varied and non-specific. Measurement of systemic saturation by pulse oximetry should be a routine part of the physical examination. Corticosteroids should be considered as a potentially beneficial therapy for EVALI, along with supportive care and anti-infectives as warranted by clinical presentation. Our case supports the notion of the use of non-invasive oxygen support for the hypoxemia associated with EVALI. Additionally, clinicians should continue to report cases of EVALI to the CDC to aid in gathering more epidemiologic information on this syndrome.
